# Patient-centered discharge summaries to support safety and individual health literacy: a double-blind randomized controlled trial in Austria

**DOI:** 10.1186/s12913-024-11183-w

**Published:** 2024-07-09

**Authors:** Christine Maria Schwarz, Magdalena Hoffmann, Christian Smolle, Andrea Borenich, Stefan Fürst, Alexandru-Cristian Tuca, Anna Katharina Holl, Markus Gugatschka, Victor Grogger, Lars-Peter Kamolz, Gerald Sendlhofer

**Affiliations:** 1 Research Unit for Safety and Sustainability in Healthcare, c/o, Division of Plastic, Aesthetic and Reconstructive Surgery, Department of Surgery , Medical University of Graz, Graz, Austria; 2 Executive Department for Quality and Risk Management, University Hospital of Graz, Auenbruggerplatz 1/3, Graz, EG A-8036 Austria; 3 Institute for Medical Informatics, Statistics and Documentation, Medical University of Graz, Graz, Austria; 4 Division of Gastroenterology and Hepatology, Department of Internal Medicine, Medical University of Graz, Graz, Austria; 5 Department of Psychiatry and Psychotherapeutic Medicine, Medical University of Graz, Graz, Austria; 6 Division of Phoniatrics, Department of Otorhinolaryngology, Medical University of Graz, Graz, Austria; 7 Department for Medical Informatics and Processes, University Hospital of Graz, KAGes, Graz, Austria

**Keywords:** Patients, Discharge summary, Quality, Content, Health literacy, Healthcare quality improvement, Patient-centered care, Patient education

## Abstract

**Background:**

To ensure a safe patient discharge from hospital it is necessary to transfer all relevant information in a discharge summary (DS). The aim of this study was to evaluate a bundle of measures to improve the DS for physicians, nurses and patients.

**Methods:**

In a double-blind, randomized, controlled trial, four different versions of DS (2 original, 2 revised) were tested with physicians, nurses and patients. We used an evaluation sheet (Case report form, CRF) with a 6-point Likert scale (1 = completely agree; 6 = strongly disagree).

**Results:**

In total, 441 participants (physicians *n* = 146, nurses *n* = 140, patients *n* = 155) were included in the study. Overall, the two revised DS received significant better ratings than the original DS (original 2.8 ± 0.8 vs. revised 2.1 ± 0.9, *p* < 0.001). Detailed results for the main domains are structured DS (original 1.9 ± 0.9 vs. revised 2.2 ± 1.3, *p* = 0.015), content (original 2.7 ± 0.9 vs revised 2.0 ± 0.9, *p* < 0.001) and comprehensibility (original 3.8 ± 1.2vs. revised 2.3 ± 1.2, *p* < 0.001).

**Conclusion:**

With simple measures like avoiding abbreviations and describing indications or therapies with fixed contents, the DS can be significantly improved for physicians, nurses and patients at the same time.

**Trial registration:**

First registration 13/11/2020 NCT04628728 at www.clinicaltrials.gov, Update 15/03/2023.

**Supplementary Information:**

The online version contains supplementary material available at 10.1186/s12913-024-11183-w.

## Key messages

1. To ensure a safe patient discharge from hospital it is necessary to transfer all relevant information in a discharge summary (DS).

2. Several issues related to the medical DS have already been identified delayed transmission of the DS to the subsequently treating physician and others, low quality or lack of information, lack of consistent formats, lack of patient understanding, and inadequate training for medical students in writing medical DS.

3. It would also be necessary that the patients understand the DS; - or at least receive therapeutically relevant care information in form of a patient-directed DS.

4. With simple measures like avoiding abbreviations and describing fixed content, the DS can be significantly improved for patients, physicians, and nurses at the same time.

5. The DS should become a patient‐centred tool ensuring that improved communication and understanding between healthcare professionals, patients and relatives succeeds.

**Supplementary Information:**

The online version contains supplementary material available at 10.1186/s12913-024-11183-w.

## Introduction

To ensure a safe patient discharge from hospital it is necessary to transfer all relevant information in a discharge summary (DS). It is known that poor-quality discharge communications, orally and written, can lead to adverse outcomes, such as preventable readmissions [1, 2]. Moreover, there can be a negative impact on further patient care and health outcomes [3, 4].

Several issues related to the medical DS have already been identified in a review such as delayed transmission of the DS to the subsequently treating physician and others, low quality or lack of information, lack of consistent formats, lack of patient understanding, and inadequate training for medical students in writing medical DS [5].

Moreover, the use of unexplained abbreviations of medical terms may influence effective communication with all involved parties (physicians, nurses, patients and relatives) and cause relevant information to go unnoticed [6].

According to Austrian law, every patient must receive a medical DS at discharge and patients are owners of their written DS [7]. Therefore, it would also be necessary that the patients understand the DS;—or at least receive therapeutically relevant care information in form of a patient-directed DS. A study by Lin et al. [8] showed, that a simple patient-directed DS at discharge significantly improved the patient’s understanding of their illness and post discharge recommendations.

As part of preliminary projects, we have been working on the topic of communication and information in healthcare and especially on the improvement of the medical DS. In this present trial (GO-SAFE – safe discharge from hospital), existing DS of a University Hospital were analysed. First, we summarized general risks regarding medical discharge information in a review. The results of this systematic literature research indicate notable risk factors relating to the medical DS [5]. Secondly, physicians (*n* = 1060) in Styria were asked which content is important to them, what contribution the current DS is making to promote the individual health literacy of patients and for whom it is important [9]. The DS should (for the most part) satisfy the needs of physicians, nurses and patients for effective discharge communication. This was also shown in preliminary survey of inpatients conducted at regular intervals in the University Hospital in Graz demonstrated that patients frequently lacked clarity about their post-discharge treatment, therapy, and medication options. Thirdly, 100 DS were systematically analysed according to their content and strengths and weaknesses [6]. We have identified a significant issue with important items being missing in the DS as comprehensive medication history and clear recommendations for future medication. Furthermore, unclear abbreviations were regularly used. Fourth, timely delivery to doctor and patient was verified.

Based on this preliminary work, we have established quality criteria for a good DS. The main objective of this double-blind, randomized, controlled trial was to evaluate measures to improve the DS for physicians, nurses, and patients as the target groups of the document.

## Methods

### Pre-trial

In a randomized, controlled, participant-blind trial we tested two conventional DS (one surgical DS, one internal medicine DS) and revised versions of these DS. Medical undergraduates were randomized into two groups (original vs. revised) and asked to assess the assigned letter for the 3 domains i) structure, ii) content, and iii) patient-friendliness. We used an evaluation sheet (Case report form, CRF) with a 6-point Likert scale (1 = completely agree; 6 = strongly disagree): The results of the CRF were compared using the Mann-Whitney-U-Test with a *p* < 0.05 being the level of significance. In total, 74 undergraduates participated in the study [10]. Based on the pre-study the main study was designed.

### Main trial

In the main trial, two randomly selected original written DS (Department of Internal Medicine – Division of Gastroenterology and Department of Surgery—Division of Plastic, Aesthetic and Reconstructive Surgery, Medical University of Graz) with a minimum length of two pages were selected by a member of an independent department (University Hospital Graz—Controlling). DS were revised with the following 5 key principles: i) short sentences, ii) no unexplained abbreviations, iii) large font size, iv) avoidance of technical terms (especially with the recommendations), and v) no more than 4 pages of length. The DS head was identical in both letters. Patient's data were blackened in the same way in both letters. The main structure in the revised DS was according to the guidelines of the final version of the *HL7 Implementation Guide for discharge letters for ELGA* [11]. Based on this HL7 Implementation Guide a DS must contain the following sub-headings:

- Reason for admission
- Discharge diagnosis, secondary diagnoses
- Diagnostic and therapeutic measures
- Recommended medication – including tradename, active substance, route of administration, prescription, and dosage
- Therapeutic recommendations
- Information on possible allergies, intolerances, and individual risk factors (e.g., miscellaneous implants)
- Summary of diagnostic findings
- Anamnesis (optional)

A detailed prescription can be found in the supplement (supplement Table 1).

**Table 1:** Demographic characteristics of participants

	**Original DS, *N*** = 224			**Revised DS, ***N* = 217		
**Characteristic**	**Physicians**, *N* = 74^a^	**Patients**, *N* = 77^a^	**Nurses**, *N* = 73^a^	**Physicians**, *N* = 72^a^	**Patients**, *N* = 78^a^	**Nurses**, *N* = 67^a^
**Gender**						
* Female*	34 (45.9%)	29 (39.2%)	68 (93.2%)	27 (37.5%)	33 (45.2%)	61 (91.0%)
* Male*	40 (54.1%)	45 (60.8%)	5 (6.85%)	45 (62.5%)	40 (54.8%)	6 (8.96%)
* Divers*	0 (0%)	0 (0%)	0 (0%)	0 (0%)	0 (0%)	0 (0%)
* Unknown*	0	3	0	0	5	0
**Where are you working?**						
* In a hospital*	66 (89.2%)	0 (NA%)	0 (0%)	55 (79.7%)	0 (NA%)	0 (0%)
* Primary care*	8 (10.8%)	0 (NA%)	0 (0%)	14 (20.3%)	0 (NA%)	0 (0%)
* In home care (home nursing,…)*	0 (0%)	0 (NA%)	34 (47.2%)	0 (0%)	0 (NA%)	33 (49.3%)
* In an nursing institution (nursing home, retirement home…)*	0 (0%)	0 (NA%)	38 (52.8%)	0 (0%)	0 (NA%)	34 (50.7%)
* Unknown*	0	77	1	3	78	0
**How long have you been working in the health sector?**						
﻿< *5 years*	16 (21.6%)	0 (NA%)	4 (5.48%)	12 (16.7%)	0 (NA%)	5 (7.46%)
﻿*5—10 years*	26 (35.1%)	0 (NA%)	12 (16.4%)	21 (29.2%)	0 (NA%)	12 (17.9%)
﻿*11—15 years*	11 (14.9%)	0 (NA%)	16 (21.9%)	9 (12.5%)	0 (NA%)	9 (13.4%)
﻿*16—20 years*	3 (4.05%)	0 (NA%)	13 (17.8%)	6 (8.33%)	0 (NA%)	14 (20.9%)
﻿> *20 years*	18 (24.3%)	0 (NA%)	28 (38.4%)	24 (33.3%)	0 (NA%)	27 (40.3%)
﻿*Unknown*	0	77	0	0	78	0
**Age in years**						
﻿*18—30 years*	0 (NA%)	9 (12.0%)	0 (NA%)	0 (NA%)	14 (18.2%)	0 (NA%)
* 31—43 years*	0 (NA%)	8 (10.7%)	0 (NA%)	0 (NA%)	11 (14.3%)	0 (NA%)
* 44—56 years*	0 (NA%)	15 (20.0%)	0 (NA%)	0 (NA%)	14 (18.2%)	0 (NA%)
* 57—69 years*	0 (NA%)	31 (41.3%)	0 (NA%)	0 (NA%)	29 (37.7%)	0 (NA%)
* 70—82 years*	0 (NA%)	12 (16.0%)	0 (NA%)	0 (NA%)	8 (10.4%)	0 (NA%)
* Older than 82 years*	0 (NA%)	0 (0%)	0 (NA%)	0 (NA%)	1 (1.30%)	0 (NA%)
* Unknown*	74	2	73	72	1	67
**Highest completed education**						
* Compulsory school leaving certificate, secondary school leaving certificate, other*	0 (NA%)	7 (10.4%)	0 (NA%)	0 (NA%)	9 (14.3%)	0 (NA%)
*Apprenticeship diploma, baccalaureate, higher vocational school*	0 (NA%)	43 (64.2%)	0 (NA%)	0 (NA%)	45 (71.4%)	0 (NA%)
*Master's degree, university degree*	0 (NA%)	17 (25.4%)	0 (NA%)	0 (NA%)	9 (14.3%)	0 (NA%)
﻿*Unknown*	74	10	73	72	15	67
**Where do you live?**						
*City (*> *50.000 inhabitants)*	0 (NA%)	20 (27.4%)	0 (NA%)	0 (NA%)	25 (34.7%)	0 (NA%)
*City (*< *50.000 inhabitants)*	0 (NA%)	53 (72.6%)	0 (NA%)	0 (NA%)	47 (65.3%)	0 (NA%)
*Unknown*	74	4	73	72	6	67

^a^n (%)

Missing content, e.g., information on allergies, was added according to the given structure. The design of re-worked DS was identical to the specifications of the University Hospital Graz to make it difficult to distinguish between the original and revised DS versions.

### Evaluation sheet (Case report form)

For the evaluation process, an evaluation sheet (CRF) with 3 main domains was used: i) structure, ii) content, and iii) patient-friendliness. Every section could be rated on a 6-point Likert scale (1 = completely agree, 6 = strongly disagree). The global indicator, along with the three main domains, is computed by averaging their respective items. Thus, the scale once again falls between 1 and 6, with lower scores indicating better outcomes. The evaluation sheet can be found in the supplement (supplement CRF).

### Study design

**Fig. 1:**
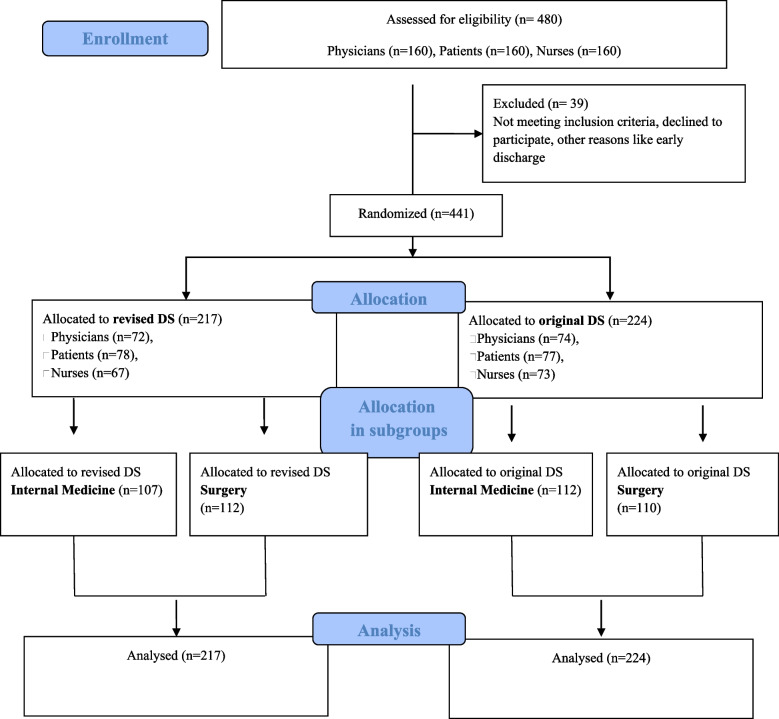
Flow diagram

The four documents (2 original DS, 2 revised DS) were tested randomly and double-blind. One of the four versions of the DS was randomly distributed to each participant in a closed envelope. Neither the participant nor the examiner knew who got which version. The target groups were physicians, nurses, and patients. Unblinding took place during the statistical analysis. A detailed description can be found in Fig. 1.

### Participants and recruitment process

Based on our pre-study it was planned to recruit a minimum of 61 participants per group over a period of 3 months (June to August 2019). If a participant agreed to take part in the study, the participant was randomly allocated to one of the four groups and received the selected version of the DS and an evaluation sheet. The evaluation sheet was returned in a sealed envelope.

### Physicians

Physicians were recruited from four different departments (Department of Surgery; Department of Internal Medicine; Department of Ear, Nose and Throat, Department of Psychiatry) at the University Hospital of Graz. The physicians received verbal information from a trained physician. Inclusion criteria were to be a trained physician and willingness to participate.

### Nurses

Nurses were recruited by the management of *Volkshilfe Steiermark*, a large nursing home institution. They received verbal information from a trained head nurse. Inclusion criteria were to be a trained nurse and willingness to participate.

### Patients

Patients were recruited from two Departments (Department of Internal Medicine – Division of Gastroenterology and Department of Surgery—Division of Plastic, Aesthetic and Reconstructive Surgery) at the University Hospital of Graz. Patients received verbal information from a trained physician as well as an informed consent form.

### Statistical methods

The items in the main domains of the survey were rated from the categories ‘completely agree’ (score 1) to ‘strongly disagree’ (score 6). Indicators, which are defined as the mean of subsets of the items, were descriptively analysed using means and standard deviations. A subgroup analysis was carried out to determine possible gender-specific differences. Differences between the two groups, the original and revised DS, were determined by t-Test. A *p*-value of < 0.05 was considered significant. All analyses were performed using R (version 3.6.1).

### Ethics statement

The Ethics Committee of the Medical University of Graz approved this study (EK 30–178 ex 17/18).

### Reporting

The research and reporting methodology followed the SQUIRE 2.0 [12] and CONSORT guidelines.

## Results

In total, 441 participants (physicians *n* = 146, nurses *n* = 140 and patients *n* = 155) were included in the study. A detailed description can be found in Table 1.

For each group, the numbers of participants were randomly assigned to the DS, allocated to groups and subgroups, and were analysed for the primary outcome.

**Fig. 2:**
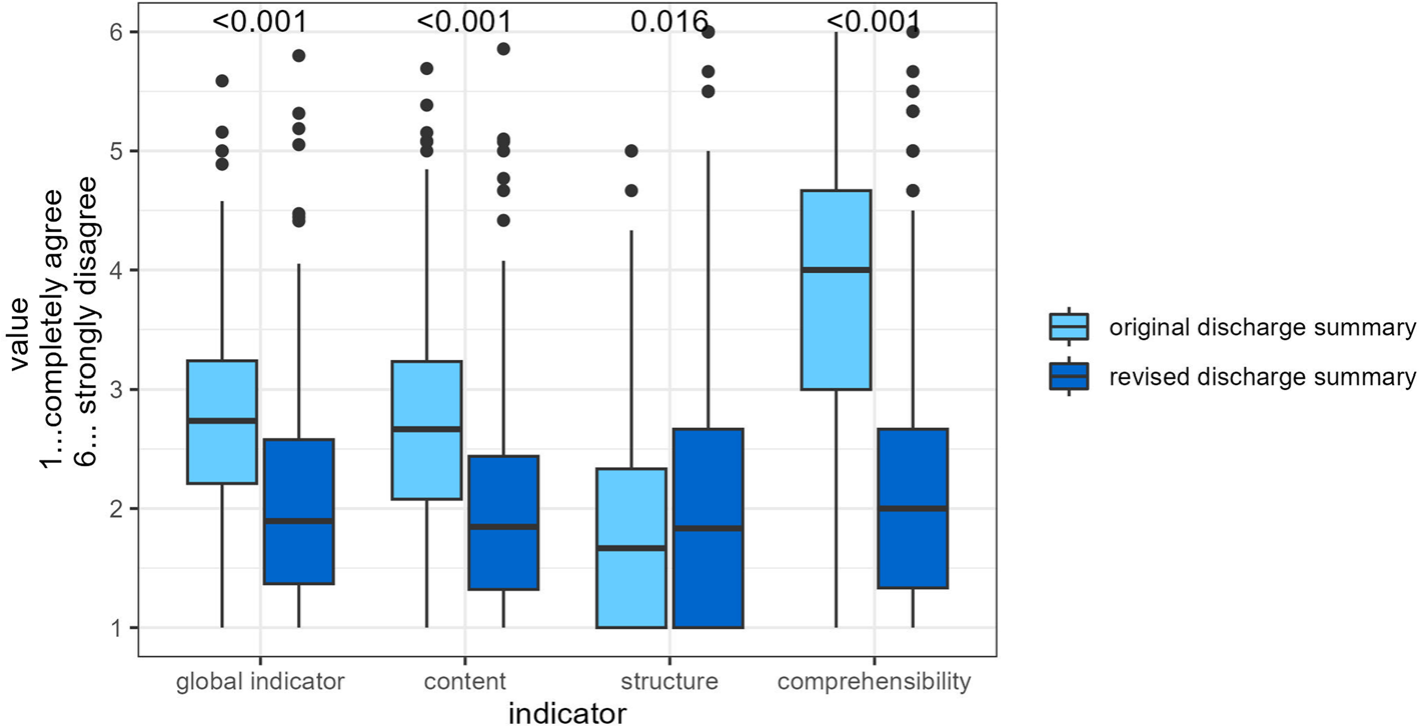
Results by indicator

Overall, the two revised DS received significant better ratings (global indicator include all domains) than the original DS (original *n* = 224; 2.8 ± 0.8 vs. revised *n* = 217; 2.1 ± 0.9, *p* < 0.001). Detailed results for the main domains were structure (original 1.9 ± 0.9 vs. revised 2.2 ± 1.3, *p* = 0.015), content (original 2.7 ± 0.9 vs. revised 2.0 ± 0.9, *p* < 0.001) and comprehensibility (original 3.8 ± 1.2 vs. revised 2.3 ± 1.2, *p* < 0.001). Details can be found in Table 2 and Fig. 2.

**Table 2:** Results by structure, content and comprehensibility

Characteristic	Original DS, *N* = 224^a^	Revised DS, *N* = 217^a^	*p*-value^b^	ES (95% CI)^c^
Global indicator	2.78 (0.80)	2.07 (0.88)	** < 0.001**	0.85 (0.65, 1.0)
Structure	1.90 (0.89)	2.15 (1.26)	**0.016**	-0.23 (-0.42, -0.04)
Content	2.74 (0.87)	2.00 (0.87)	** < 0.001**	0.85 (0.65, 1.0)
Comprehensibility	3.84 (1.20)	2.27 (1.20)	** < 0.001**	1.3 (1.1, 1.5)

^a^Mean (SD)

^b^Welch Two Sample t-test

^c^Cohen's D (95% CI)

### Results by groups

#### Physicians

Physicians rated the global indicator for the original “surgery” DS (2.6 ± 0.7) significantly worse than the revised “surgery” DS (2.2 ± 0.7). Physicians rated the global indicator for the original “internal medicine” DS (2.6 ± 0.7) and for the revised “internal medicine” DS (2.4 ± 0.9) almost equally. A striking difference was the topic of structure in the new version of “surgery” DS (original 1.9 ± 1.0 vs. revised 2.7 ± 1.3) and “internal medicine” DS (original 1.8 ± 0.9 vs. revised 3.1 ± 1.5).

### Nurses

Nursing staff rated the global indicator of the original “surgery” DS (2.7 ± 0.7) significantly worse than the revised “surgery” DS (2.0 ± 1.0). Nurses rated the global indicator of the original “internal medicine” DS (2.8 ± 0.7) significantly worse than the revised “internal medicine” DS (1.7 ± 0.7).

### Patients

Patients rated the global indicator of the original “surgery” DS (2.7 ± 0.8) significantly worse than the revised “surgery” DS (2.0 ± 0.70). Patients rated the global indicator of the original “internal medicine” DS (3.2 ± 1.0) significantly worse than the revised “internal medicine” DS (2.1 ± 1.2).

**Fig. 3:**
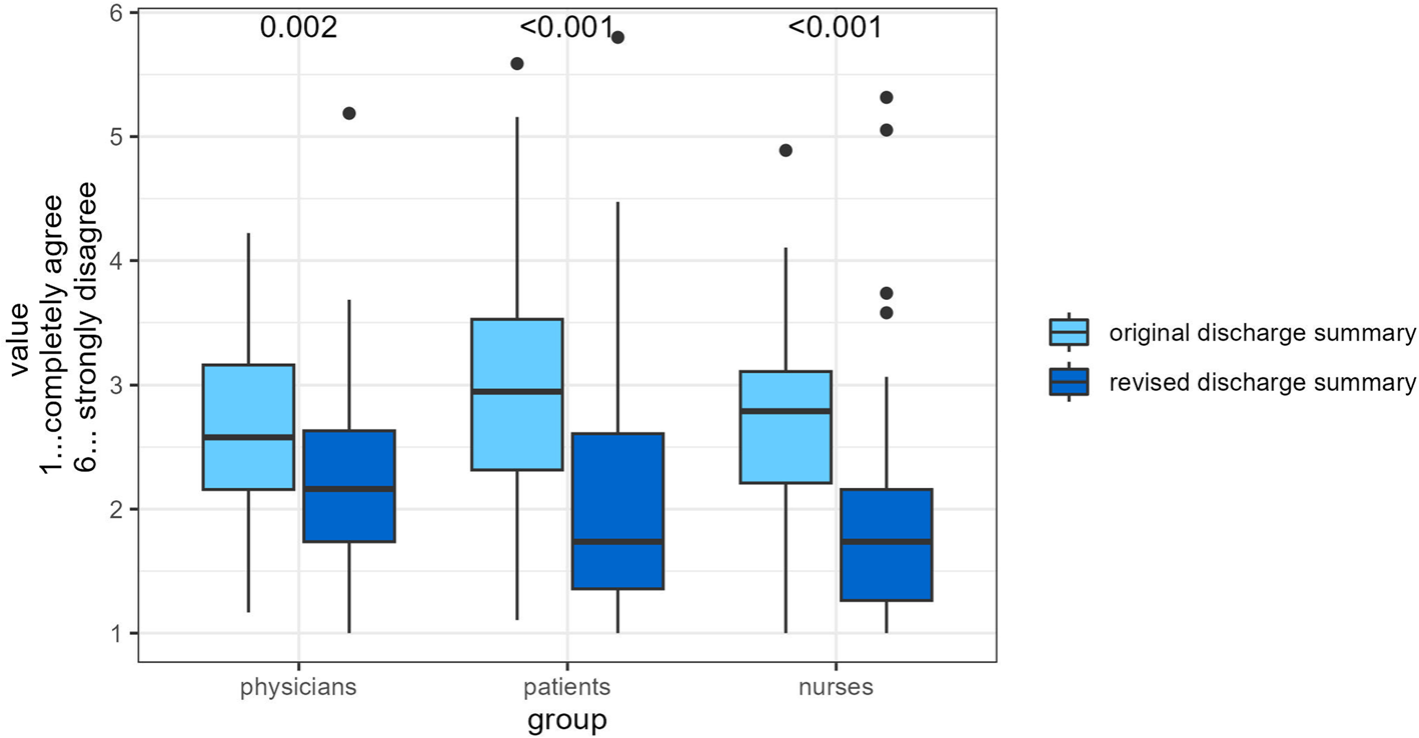
Results by groups

An overview can be found in Table 3 and Fig. 3.

**Table 3:** Results by group

	Physicians, *N* = 146					Patients, *N* = 155					Nurses, *N* = 140				
Characteristic	*N*	Original DS *N* = 74^a^	Revised DS, *N* = 72^a^	*p*-value^b^	ES (95% CI)^c^	*N*	Original DS, *N* = 77^a^	Revised DS, *N* = 78^a^	*p*-value^b^	ES (95% CI)^c^	*N*	Original DS, *N* = 73^a^	Revised DS, *N* = 67^a^	*p*-value^b^	ES (95% CI)^c^
Global indicator	145	2.61 (0.69)	2.24 (0.74)	**0.002**	0.51 (0.18, 0.85)	155	3.00 (0.92)	2.07 (0.98)	** < 0.001**	0.98 (0.65, 1.3)	140	2.71 (0.71)	1.88 (0.87)	** < 0.001**	1.1 (0.70, 1.4)
Structure	145	1.87 (0.94)	2.88 (1.37)	** < 0.001**	-0.85 (-1.2, -0.51)	154	2.05 (0.95)	1.85 (1.10)	0.2	0.19 (-0.13, 0.51)	137	1.77 (0.74)	1.71 (0.92)	0.7	0.08 (-0.26, 0.41)
Content	144	2.56 (0.70)	2.06 (0.71)	** < 0.001**	0.71 (0.38, 1.1)	154	3.06 (1.00)	2.09 (0.98)	** < 0.001**	0.97 (0.64, 1.3)	139	2.60 (0.80)	1.83 (0.88)	** < 0.001**	0.90 (0.55, 1.3)
Comprehensibility	142	3.56 (1.13)	2.31 (1.01)	** < 0.001**	1.2 (0.81, 1.5)	152	3.80 (1.27)	2.19 (1.28)	** < 0.001**	1.3 (0.90, 1.6)	138	4.15 (1.14)	2.31 (1.29)	** < 0.001**	1.5 (1.1, 1.9)

^a^Mean (SD)

^b^Welch Two Sample t-test

^c^Cohen*’*s D (95% CI)

### Results by gender

Female participants (global indicator) rated the original DS (2.8 ± 0.8) and the revised DS (2.0 ± 0.9) DS. Male participants (global indicator) rated the original DS (2.8 ± 0.8) and the revised (2.2 ± 0.8) DS.

**Fig. 4:**
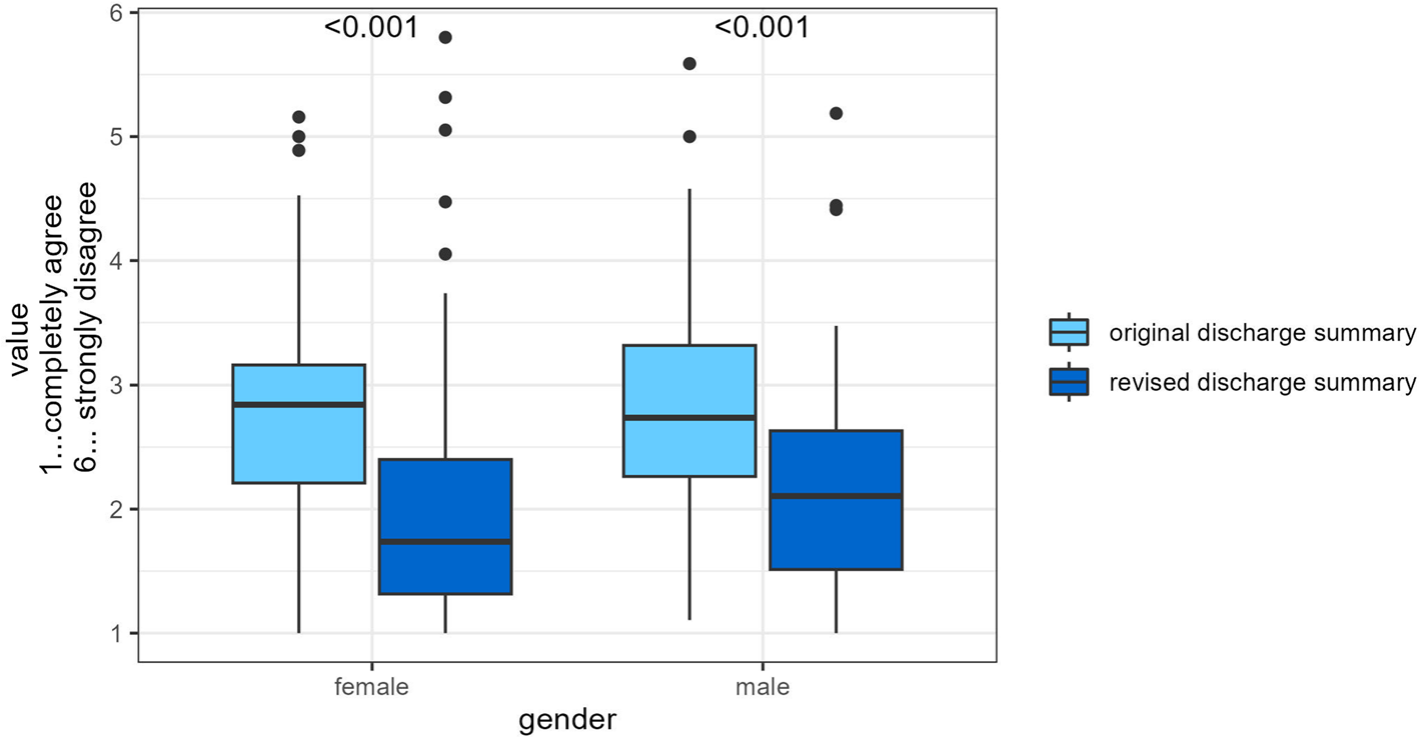
Results by gender

An overview can be found in Table 4 and Fig. 4.

**Table 4:** Results by gender

	Female, *N* = 252					Male, *N* = 181				
Characteristic	*N*	Original DS, *N* = 131^a^	Revised DS, *N* = 121^a^	*p*-value^b^	ES (95% CI)^c^	*N*	Original DS, *N* = 90^a^	Revised DS, *N* = 91^a^	*p*-value^b^	ES (95% CI)^c^
Global indicator	252	2.78 (0.77)	2.01 (0.93)	** < 0.001**	0.91 (0.65, 1.2)	180	2.78 (0.84)	2.17 (0.80)	** < 0.001**	0.73 (0.43, 1.0)
Structure	249	1.86 (0.92)	2.03 (1.26)	0.2	-0.15 (-0.40, 0.10)	179	1.96 (0.85)	2.37 (1.26)	**0.012**	-0.38 (-0.68, -0.08)
Content	251	2.74 (0.84)	1.95 (0.93)	** < 0.001**	0.90 (0.63, 1.2)	178	2.74 (0.92)	2.08 (0.79)	** < 0.001**	0.76 (0.46, 1.1)
Comprehensibility	248	3.87 (1.17)	2.28 (1.32)	** < 0.001**	1.3 (1.0, 1.6)	176	3.80 (1.25)	2.30 (1.04)	** < 0.001**	1.3 (0.97, 1.6)

^a^Mean (SD)

^b^Welch Two Sample t-test

^c^Cohen's D (95% CI)

## Discussion

Overall, the study evaluated a bundle of measures to improve the DS for physicians, nurses and patients. The findings of the evaluation can be considered an expression of the opinions of the respective groups with their own experiences. It is worth noting that, to date, no comparable study has been conducted using the same methodology. In this trial we found out that the revised DS reached significantly better ratings than the original ones.

Results showed that the original DS received already good ratings, but with simple measurements, like short sentences, no use of unexplained abbreviations, large font size, avoidance of technical terms (especially within the recommendations for health-promoting behaviour) in a standardized structure with no more than 4 pages length there could be a significant improvement. Beside these formal aspects it is also important to ensure that the discharge summary is given to the patient at discharge and that it is sent at the appropriate time to the further treating physician. Moreover, the importance of this topic should be highlighted in education and training.

The Patient Oriented Discharge Summary (PODS) is an individualized discharge tool designed with patient and family engagement that contains five sections of information that are actionable and useful for patients and families [13].

Patients, as the primary owners of their DS, rated the revised version significantly better. Since many people in Austria have a mediocre health literacy, it seems particularly important to formulate certain contents of the DS, such as instructions and further recommendations, in a way that is particularly comprehensible to the patients [14]. To communicate “about” rather than “with” patients still reflects historical behavior but is no longer deemed appropriate [9].

The evaluation of the DS was not uniform in all areas. Physicians rated the new structure in the DS significantly worse than in the original DS. Prior to the implementation of the electronic fever chart and standardised structure, the different clinics had employed varying formats for discharge summaries in Austria. General changes in the usual structure were sometimes not perceived as pleasant. However, the universal structure of the DS is specified by ELGA and now mandatory.

Nurses rated the revised DS significantly better as well. A good transfer of knowledge and information to the nursing staff is in general particularly relevant because nurses care for patients in their homes or nursing homes.

A future recommendation could therefore be, that all professional groups have a joint DS [15]. Poor information and communication between the professional groups, patients and relatives during the discharge process is a risk to patient safety, and further research could investigate this impact. As far as possible, the DS should be adapted to the needs of the addressees. Great attention must be paid to the use of resources. Electronic documentation and Artificial Intelligence can contribute to this [16–18].

In practice there are examples of “*own*” patient DS [8, 19, 20]. The DS should become a patient‐centred tool ensuring that improved communication and understanding between healthcare professionals, patients and relatives succeeds. Many positive effects, such as increased patient satisfaction, increased understanding and a greater patient involvement, were shown [21]. This should be achieved without reducing the medically relevant content.

## Strengths and limitations of the study

A strength of this study was the design of the trial as all stakeholders namely patients, physicians, and nurses were involved. The newly developed DS can support them all at the same time. However, the study does not allow to verify whether the revised discharge letters actually contribute to better patient care.

There are some limitations in this study. First, the physicians may have noticed that there are differences to the letters from their own department. However, this bias is negligible since each University hospital has different formats and structures at the University Hospital of Graz. Basically, only the letterhead was uniform across the clinic. Secondly, in this study, we only examined and revised two randomly selected DS.

## Conclusions

The aim of this study was to evaluate a bundle of measures to improve the DS for physicians, patients, and nurses. It can be stated that across all groups, the revised version of the DS received significant better ratings by all participants. Only the item of structure, which is mandatory by ELGA regulations, received a poorer rating in the new version exclusively from the physicians surveyed. To summarize, small improvements can make a difference for all groups at the same time.

### Supplementary Information

Supplementary Material 1.

Supplementary Material 2. Evaluation’s sheet.

## Data Availability

The datasets used and/or analyzed during the current study are available from the corresponding author on reasonable request. Contact details: Magdalena Hoffmann. Medical University of Graz, Auenbruggerplatz 29/4, 8036 Graz, Austria, Email: Magdalena.hoffmann@medunigraz.at, Telephone: + 43 316 385 80,804.

## Abbreviations

DS: Discharge Summary
e.g.: Exempli gratia
ELGA: “Elektronische Gesundheitsakte” – Austrian Electronic Health Records
IQR: Interquartile ranges
CRF: Case Repot Form
SQUIRE: Standards for Quality Improvement Reporting Excellence
PODS: Patient Oriented Discharge Summary
IT: Information Technology
