# Supplementary material for: Patient-centered discharge summaries to support safety and individual health literacy: a double-blind randomized controlled trial in Austria
Source: BMC Health Serv Res. 2024 Jul 9;24:789. doi: 10.1186/s12913-024-11183-w (PMC11234775; doi:10.1186/s12913-024-11183-w)
Supplement: Supplementary file 1 — Supplementary Material 1. [file 12913_2024_11183_MOESM1_ESM.docx]

*Supplement Table 1: Mandatory and optional ELGA headings of the medical DS*

| Option | Position | Section | | |
| --- | --- | --- | --- | --- |
| *[O]* | 1 | Letter text | | |
| *[M]* | 2 | **Reason for admission** | | Epicrisis |
| *[M]* | 3 | **Diagnosis at discharge** | |  |
| *[O]* | 4 | Rehabilitation goals | |  |
| *[O]* | 5 | Outcome Measurement | |  |
| *[O]* | 6 | Measures implemented | |  |
| *[M]* | 7 | **Last medication** | |  |
| *[M]* | 8 | **Recommended medication** | |  |
| *[M]* | 9 | **Further recommended measures** | |  |
|  |  | ***[R2]*** | **Appointments, control** |  |
|  |  | ***[R2]*** | **Discharge condition** |  |
|  |  | ***[R2]*** | **Recommended arrangements for further care** |  |
| *[O]* | 10 | Summary of stay | |  |
| *[O]* | 11 | Closing remarks | | |
| *[R2]* | 12 | Allergies, intolerances and risks | | Secondary Sections |
| *[O]* | 13 | Diagnostic findings  Possible Subsections: | |  |
|  |  | *[R2]* | Pending results |  |
|  |  | *[R2]* | Extracts from collected results |  |
|  |  | *[R2]* | Operation report |  |
|  |  | *[R2]* | Attached collected results |  |
|  |  | *[R2]* | Vital parameters |  |
| *[O]* | 14 | Anamnesis | |  |
| *[O]* | 15 | Previous diseases | |  |
|  |  | *[O]* | Subsection “Previous measures” |  |
| *[O]* | 16 | Medication at admission | |  |
| *[O]* | 17 | Medication administered during the stay | |  |
| *[O]* | 18 | Living wills and other legal documents | |  |
| *[O]* | 19 | Supplements | |  |

Legend: MUST means a mandatory requirement (commandment). Corresponds to the conformity criteria [R] and [M]. SHOULD or RECOMMENDED stands for a recommendation. It is desired and recommended that the requirement should be implemented, but there may be reasons why this is not done. Corresponds to compliance criterion [R2]. CAN or OPTIONAL (MAY, OPTIONAL): The implementation of the requirement is optional, it can also be omitted without compelling reason. Corresponds to the conformity criterion [O].
