# Supplementary material for: Patient-centered discharge summaries to support safety and individual health literacy: a double-blind randomized controlled trial in Austria
Source: BMC Health Serv Res. 2024 Jul 9;24:789. doi: 10.1186/s12913-024-11183-w (PMC11234775; doi:10.1186/s12913-024-11183-w)
Supplement: Supplementary file 2 — Supplementary Material 2. Evaluation’s sheet. [file 12913_2024_11183_MOESM2_ESM.pdf]

University Hospital of Graz

Evaluation is carried out by the Department für Quality-and Riskmanagement

Mark as shown: ☐ ☒ ☐ ☐ ☐ Please use a ball-point pen or a thin felt tip. This form will be processed automatically.

Correction: ☐ ■ ☐ ☒ ☐ Please follow the examples shown on the left hand side to help optimize the reading results.

1. General Information

Dear Sir or Madam

This survey analyses the content and structure of the current medical discharge summary. We are interested in your opinion. Your answers will contribute to the further development of the discharge summary. Participation in this research project is voluntary. Participants can withdraw at any time, even after the beginning of the survey, and do not have to give any reasons for doing so. Refusal to participate or early withdrawal will have no negative consequences for you. No personal data will be requested. It is therefore not possible to draw any conclusions about you as an individual. If you have any questions, please contact PD. Dr Gerald Sendlhofer (gerald.sendlhofer@klinikum-graz.at).

Thank you very much!

1.1 Your sex: ☐ Female ☐ Male ☐ Diverse

1.2 Where do you work? ☐ In a hospital ☐ in general practice

1.3 If you work in a hospital, what position do you have? (multiple selections possible) ☐ junior physician ☐ ward physician ☐ assistant physician ☐ specialist physician ☐ senior physician ☐ leading function

1.4 If you work in the general practice setting, you are… ☐ a specialist in general medicine ☐ specialist for other subjects

1.5 How long have you been working in the healthcare sector? ☐ <5 years ☐ 5 - 10 years ☐ 11-15 years ☐ 16-20 years ☐ >20 years

2. Structure of the discharge summary

		1 (Totally agree)	2	3	4	5	6 (disagree at all)
2.1	Is the layout clear?	☐	☐	☐	☐	☐	☐
2.2	Is the structure clear (logical)?	☐	☐	☐	☐	☐	☐
2.3	In your opinion, does the content of the document justify its length?	☐	☐	☐	☐	☐	☐

2.4 Do you have any comments on the structure of the discharge summary?

3. Content of the discharge summary

		1 (Totally agree)	2	3	4	5	6 (disagree at all)
3.1	Are the abbreviations used clear?	☐	☐	☐	☐	☐	☐
3.2	Are the abbreviations used explained?	☐	☐	☐	☐	☐	☐

3. Content of the discharge summary [Continue]

		1 (Totally agree)	2	3	4	5	6 (disagree at all)
3.3	Is the discharge summary generally formulated in a way that you can understand?	☐	☐	☐	☐	☐	☐
3.4	Is the chronological sequence of events during the inpatient stay presented coherently?	☐	☐	☐	☐	☐	☐
3.5	Are the main and secondary diagnoses clearly stated?	☐	☐	☐	☐	☐	☐
3.6	Are there comprehensible reasons for a treatment measure taken in the hospital?	☐	☐	☐	☐	☐	☐
3.7	In your opinion, are the further therapy recommendations or rehabilitation goals presented in a comprehensible and comprehensive manner?	☐	☐	☐	☐	☐	☐
3.8	Are the measures taken comprehensible and comprehensively presented?	☐	☐	☐	☐	☐	☐
3.9	Are the clinical conditions (including diagnoses) for the recommended medication adequately presented?	☐	☐	☐	☐	☐	☐
3.10	Is the recommended medication clearly described (name, active substances, dosage, method of administration...)?	☐	☐	☐	☐	☐	☐
3.11	Can you derive the next necessary steps and measures/controls for further treatment/care from the discharge summary?	☐	☐	☐	☐	☐	☐
3.12	Can you find information on possible allergies?	☐	☐	☐	☐	☐	☐
3.13	Can you find the contact address of a physician for further enquiries?	☐	☐	☐	☐	☐	☐

3.14 Are you missing important information in the discharge summary or do you have any comments?

4. Patient comprehensibility of the discharge summary

		1 (Totally agree)	2	3	4	5	6 (disagree at all)
4.1	Would a medical layperson understand the contents of the discharge summary?	☐	☐	☐	☐	☐	☐
4.2	Would a medical layperson be able to understand the prescribed medication (application area)?	☐	☐	☐	☐	☐	☐
4.3	Would a medical layperson be able to extract the next necessary measures and steps from the discharge summary?	☐	☐	☐	☐	☐	☐
4.4	Is there anything else you would like to tell us about the discharge summary?	☐	☐	☐	☐	☐	☐

Thank you for your co-operation!
